# Pre‐Existing and Gestational Diabetes and Risk of Maternal Venous Thromboembolism: A Systematic Review and Meta‐Analysis of Observational Studies

**DOI:** 10.1111/1471-0528.18043

**Published:** 2024-12-17

**Authors:** Molly Orrin, Emilia Barber, Matthew J. Grainge

**Affiliations:** ^1^ Academic Unit of Lifespan and Population Health, School of Medicine University of Nottingham Nottingham UK

**Keywords:** antepartum, diabetes, postpartum, systematic review, venous thromboembolism

## Abstract

**Background:**

Women who are pregnant are at increased risk of venous thromboembolism (VTE), which persists for up to 3 months following childbirth. Diabetes is known to increase the risk of serious cardiovascular outcomes.

**Objective:**

To comprehensively review literature on the extent to which pre‐existing or gestational diabetes influences the risk of VTE in both pregnancy and postpartum.

**Search Strategy:**

We used Medline, Embase and Google Scholar to identify observational studies published up to 2 November 2023.

**Selection Criteria:**

Studies which quantified the relationship between diabetes on antepartum and/or postpartum VTE, and which provide separate data for pre‐existing and gestational diabetes.

**Data Collection and Analysis:**

Results were pooled, where appropriate, using random‐effects meta‐analysis.

**Main Results:**

Twenty one studies from Europe, the United States and Asia were included. There was an increased risk of antepartum VTE in women with gestational diabetes (RR = 2.48, 95% CI 1.47 – 4.16, *I* ^2^= 45%, 4 studies) but not pre‐existing diabetes (RR = 1.71, 0.43 – 6.77, *I* ^2^= 68%, 2 studies). For postpartum VTE, there was no clear association with either pre‐existing (RR = 1.28, 0.73 – 2.24, *I* ^2^= 73%, 6 studies) or gestational (RR = 1.39, 0.77 – 2.51, *I* ^2^= 70%, 10 studies) diabetes.

**Conclusions:**

Our results will provide some reassurance for pregnant women with pre‐existing or gestational diabetes, owing to no clear evidence of an increased risk of maternal VTE. While some studies report a raised risk of VTE during antepartum specifically, results must be interpreted in light of high levels of heterogeneity.

## Abstract 2

**Linked article:** This article is commented on by van der Slink pp. 1086 in this issue. To view this article visit https://doi.org/10.1111/1471-0528.18138.

## 1 Introduction

Venous thromboembolism (VTE) remains the leading direct cause of maternal mortality during or up to 6 weeks after the end of pregnancy in the United Kingdom, accounting for over 10% of maternal deaths [1]. When nonfatal, complications from VTE include severe postthrombotic syndrome in approximately 5%–10% of cases, [2] while in all cases there is the need for medium‐term anticoagulation. Overall, VTE affects around 1–2 in every 1000 pregnancies, and an increased risk of VTE is likely to persist for up to 3 months following childbirth [3, 4, 5].

Current guidance from the UK Royal College of Obstetricians and Gynaecologists (RCOG) includes an algorithm to identify which women should receive thromboprophylaxis to prevent VTE in and around pregnancy [6]. These state that all women admitted to hospital when pregnant should be considered for thromboprophylaxis with low‐molecular‐weight heparin (LMWH), while those women with two or more risk factors at the time of delivery should be considered for LMWH for at least 10 days postpartum. A risk prediction model provided a more refined estimate of which women were at highest risk of developing postpartum VTE using UK healthcare data [7]. In this model, medical comorbidities were grouped into a single entity, thus failing to adequately consider prophylaxis strategies for specific medical conditions.

Diabetes causes physiological changes such as altered concentrations of coagulatory proteins, elevated levels of triglycerides and low levels of high‐density lipoprotein. These physiological changes can contribute to hypercoagulability and hence an increase in the risk of cardiovascular diseases such as VTE [8]. In the United States and Europe, it is estimated that around 10% of pregnancies are affected by gestational diabetes [9, 10]. A recent value of information study concluded that future research on this topic should focus on randomised trials of prophylaxis effectiveness in obese postpartum women with additional risk factors [11]. Obesity is linked to diabetes and therefore a plausible reason as to why obesity is a risk factor for maternal VTE. As such, the presence of diabetes could be considered a separate criterion for identifying women to participate in clinical trials. However, the degree to which diabetes increases the risk of VTE or whether any association is due to confounding by other variables which directly influence maternal VTE is not fully understood.

In this systematic review, we synthesised population‐based research on the association between diabetes and maternal VTE, primarily exploring whether there were differences between pre‐existing and gestational diabetes and whether the association differed for VTE occurring during pregnancy or postpartum.

## 2 Methods

This review followed the Preferred Reporting Items for Systematic Reviews and Meta‐Analyses (PRISMA) checklist for systematic reviews [12]. The review protocol was registered with PROSPERO (Protocol No. CRD42022352967).

### 2.1 Inclusion Criteria

#### 2.1.1 Population

Studies assessing risk of VTE during pregnancy or following childbirth. No restrictions were made according to age. Where participants were from the same source as the population used in an existing study, additional papers were also eligible for inclusion if they provided data for a separate meta‐analysis.

#### 2.1.2 Exposure

Pre‐existing and gestational diabetes. Studies were excluded if these were combined into a single measure of ‘diabetes’ as not being able to separate pre‐existing from gestational diabetes would limit the impact of results on clinical practice.

#### 2.1.3 Comparator

Women without pre‐existing or gestational diabetes.

#### 2.1.4 Outcome

Symptomatic VTE, either deep vein thrombosis or pulmonary embolism, during pregnancy or postpartum. We included studies which used electronic health data if medical codes used to identify VTE events were provided.

#### 2.1.5 Study design and reporting

We included case–control, cross‐sectional and cohort designs. We excluded randomised trials, case reports, case series and review articles, in addition to any studies published only as conference abstracts (no available full text). Only papers published in English were considered, but no restriction was made according to year of publication.

### 2.2 Search Strategy

An updated literature search was run on 2 November 2023. This was performed using two bibliographic databases MEDLINE (OVID 1946 to present) and EMBASE (OVID 1974 to present). We also used Google Scholar as part of the primary search, including the first 300 results when the review question was entered into the search bar, irrespective of relevance. The full search strategy is provided in Appendix S1. The sensitivity of the search was checked using an existing collection of nine articles deemed relevant held by one of the authors (MJG). As all these articles were picked up by the primary search, no modification of the search strategy was needed.

### 2.3 Study Selection

Records picked up by the search were imported into either Rayyan (initial search) or Covidence (updated search). All titles and abstracts were independently screened by two authors (either MO/MJG or EB/MJG) with any conflicts resolved through discussion. Full texts were reviewed for inclusion by the same pairs of researchers.

### 2.4 Data Extraction

Data extraction was carried out by a single author (MO) and checked by a second (MJG). For each included paper, study details (author(s) and publication year), study characteristics (design and country), participant data (study size, age and postpartum duration), exposure detail (pre‐existing or gestational diabetes), outcome detail (type of VTE and ascertainment of VTE diagnosis) and which variables were adjusted for (confounders) were extracted using piloted data forms.

For each exposure/outcome association, we extracted the natural log of the risk estimate (hazard, risk or odds ratio) and its standard error. Where results were presented from multiple models, the most fully adjusted result was extracted. If only raw numbers presented in a 2x2 table were available, we used standard formula to estimate the risk/odds ratio and standard error [13]. Where possible, we extracted numerical data from the entire cohort rather than subgroups of participants. However, Tepper et al. [14] reported separate results for women with private insurance and women with Medicaid (for both pre‐existing and gestational diabetes). These results were pooled using fixed effects to provide a result for a single population for use in subsequent meta‐analyses.

### 2.5 Risk of Bias (RoB) Assessment

Studies were assessed for RoB using the Newcastle–Ottawa scale (NOS) [15]. This evaluates papers according to three criteria: selection, comparability and either ascertainment of outcome (for cohort and cross‐sectional studies) or ascertainment of exposure (for case–control studies). This resulted in a score between 0 and 9, where higher scores indicate higher quality (or lower RoB). Further details of the tool for each study design are provided in Appendix S2. For comparability, a total of 2 points were awarded. One point was awarded when the study adjusted for maternal age as age is frequently treated as a forced‐in variable for epidemiological regression models owing to its role as a potential confounder in most situations [16]. A second point was awarded if they adjusted for any variable listed in algorithm used in the RCOG guideline as identifying patients as intermediate risk for antepartum or postpartum prophyalxis [6]. All RoB assessment was carried out by two authors (MO and MJG) with any conflicts resolved through discussion.

### 2.6 Data Synthesis and Assessment of Heterogeneity

Meta‐analyses were performed separately for pre‐existing and gestational diabetes and for antepartum and postpartum periods, assuming separate data for these groups/time periods were available. Where only data on VTE risk during antepartum and postpartum combined (the entire maternity) were available, these were included in a separate analysis. Results were pooled using the generic inverse‐variance method, assuming random effects. The Hartung–Knapp–Sidik–Jonkman method was used to estimate the between‐study variance. This has been shown to provide more reliable results for random‐effects meta‐analysis than the DerSimonian and Laird approach when the number of studies is small and there is moderate‐to‐substantial heterogeneity [17, 18].

Heterogeneity was quantified using the *I* ^2^ statistic, with values > 70% representing considerable heterogeneity [19]. Studies using different effect measures (e.g., odds and incident rate ratios) were pooled in the same analysis but presented as separate subgroups. No other subgroup or sensitivity analyses were predefined in the study protocol but in a post hoc analysis, we repeated all meta‐analyses with only studies that adjusted for obesity in recognition that this variable is likely to be strongly related to diabetes and a recent health economic study which concluded that targeting this group for future clinical trials is most likely to be cost effective [11]. In an additional protocol deviation, we extended the postpartum duration from 6 weeks in the published protocol to 6 months in acknowledgement that the original interval was too restrictive and to rely instead on the authors' definition of postpartum duration.

Funnel plots and tests for publication bias were not presented as all meta‐analyses contained 10 or fewer studies. All analysis was carried out using the ‘meta’ package in R.

### 2.7 Patient and Public Involvement

Patients and members of the public were not engaged in the development of this study.

## 3 Results

### 3.1 Overview of Search Results

**FIGURE 1:**
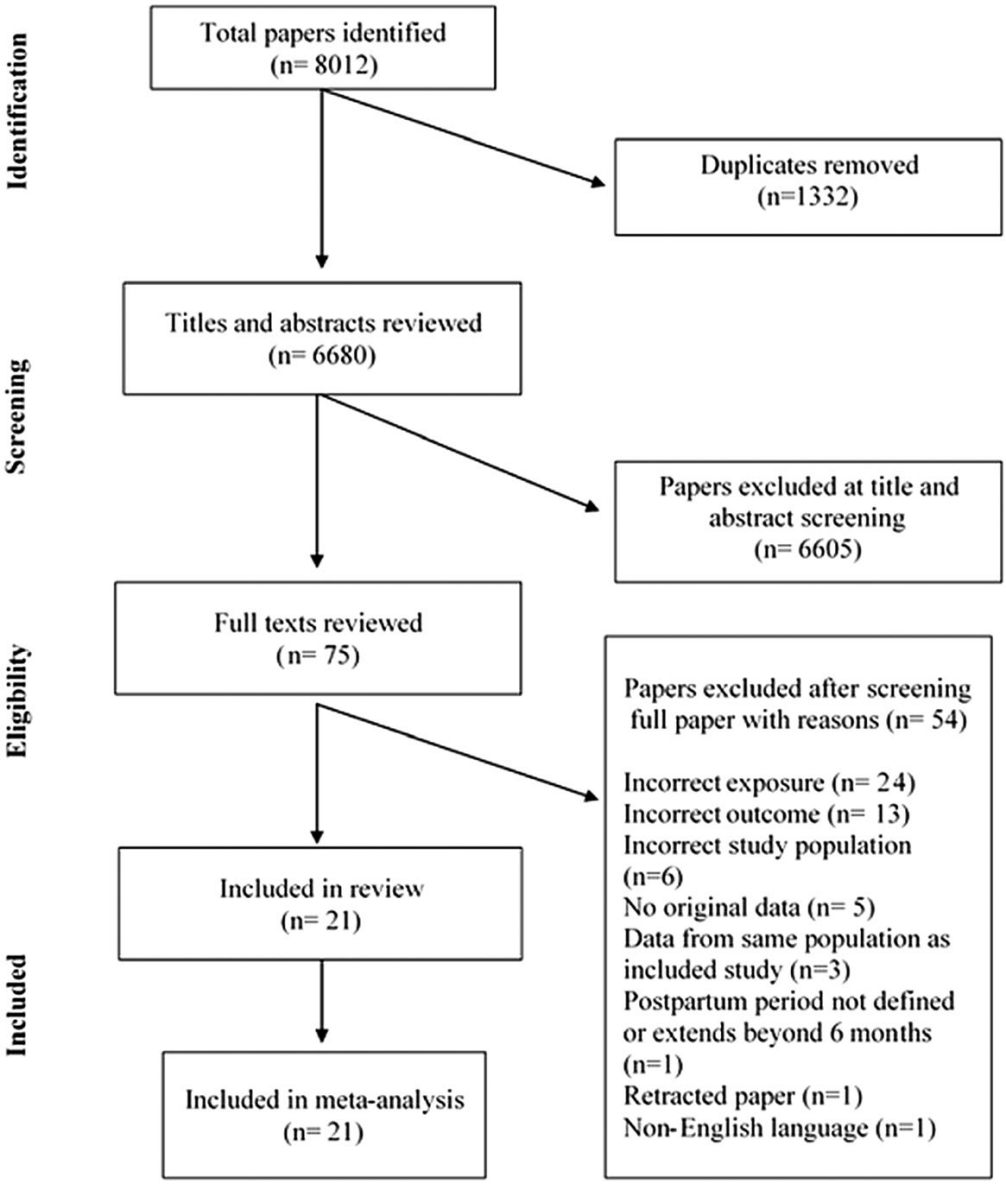
PRISMA flow diagram of included and excluded studies.

Our search yielded a total of 6680 papers after removal of duplicates, of which 75 were screened as full texts and 21 papers were included in the review (Figure 1) [14, 20, 21, 22, 23, 24, 25, 26, 27, 28, 29, 30, 31, 32, 33, 34, 35, 36, 37, 38, 39]. Main reasons for exclusion at the full stage were due to the exposure variable (*n* = 24), including studies which only presented data for pre‐existing and gestational diabetes combined and due to the outcome variable (*n* = 13), including failure to provide data for venous thrombotic events specifically. Two studies from Sultan and colleagues [32, 34] used data from the CPRD but were included as separate studies as they contributed data to different meta‐analyses. Similarly, registry data from Norway [25, 26] and Denmark [27, 35] were included as separate studies for the same reason. Papers by Krenitsky [28] and Wen [36] both used data from the Nationwide Readmissions Database. These studies provided different ways of defining postpartum (Table S1). Krenitsky was used in the primary analysis as this study included the greater number of study participants but in a sensitivity analysis for pre‐existing diabetes during the postpartum, we explored whether use of data from the Wen paper, altered findings. A third paper from Bleau [20] using the same database was included separately as this provided data on the combined antepartum and postpartum period. All other included studies contributed to at least one meta‐analysis. Cohort and cross‐sectional studies provided data on a total of 93 705 220 participants (median per study = 572 591), while case–control studies provided data on a total of 2021 VTE cases (median per study = 244) and 627 472 controls (median per study = 999).

### 3.2 Overview of Included Studies

Eight studies were conducted in Europe (three United Kingdom, two Norway, two Denmark and one Finland), five in the United States, seven in Asia (five China, one South Korea and one Israel) and one in Australia (Table S1). Eight studies used a case–control design, eight were cohort studies and five cross‐sectional. Fifteen studies used administrative health datasets for their analysis, while six were conducted in healthcare settings. Two included data only on antepartum women, 10 provided data on postpartum only, while 9 provided data on both antepartum and postpartum VTE. Gestational diabetes alone was considered as an exposure variable in 11 studies, 4 studies considered pre‐existing diabetes alone and 6 studies included both.

### 3.3 Methodological Quality of Included Studies

Overall quality of the studies included in the review was high with all studies comprising participants who were representative of all pregnant/postpartum women, and where data capture methods and follow‐up duration allowed all VTE events to be captured (Table S2). The main areas where studies were rated less well included failure to exclude women with a previous VTE (11 of 21 studies did not report this exclusion) and lack of further validation of VTE events (8 of 21 studies did not provide further evidence of VTE). In terms of comparability, 12 studies adjusted for a measure of age which we considered the most important confounder, while 11 adjusted for additional variables currently considered in the algorithm for prophylaxis used by the RCOG (antenatal or postnatal). Table S3 provides a complete list of all variables adjusted for in the result we extracted from each study.

### 3.4 Results: Antepartum VTE

**FIGURE 2:**
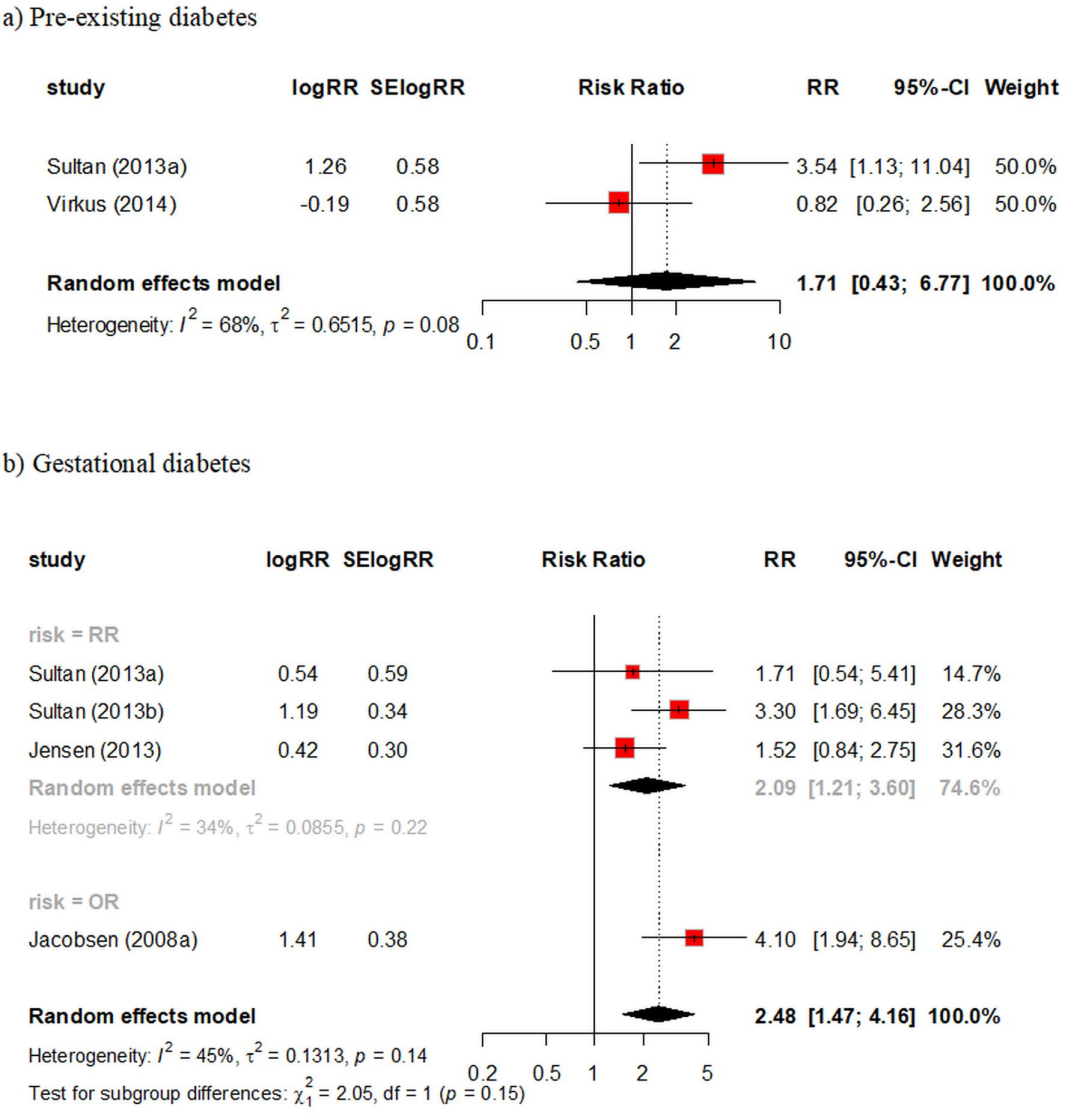
Diabetes and the risk of antepartum VTE. (a) Pre‐existing diabetes and (b) gestational diabetes.

Two studies using administrative healthcare data from the United Kingdom and Denmark provided data for pre‐existing diabetes (Figure 2). There was considerable variation in results (*I* ^2^= 68%), with a pooled RR of 1.71 (95% CI, 0.43 – 6.77).

Four studies using administrative healthcare data from the United Kingdom, Denmark and Norway provided data for gestational diabetes (Figure 2). When results were pooled, there was a 2.5‐fold increase in risk of antepartum VTE in women with gestational diabetes with some between‐study heterogeneity (RR = 2.48, 95% CI 1.47 – 4.16, *I* ^2^= 45%).

### 3.5 Results: Postpartum VTE

**FIGURE 3:**
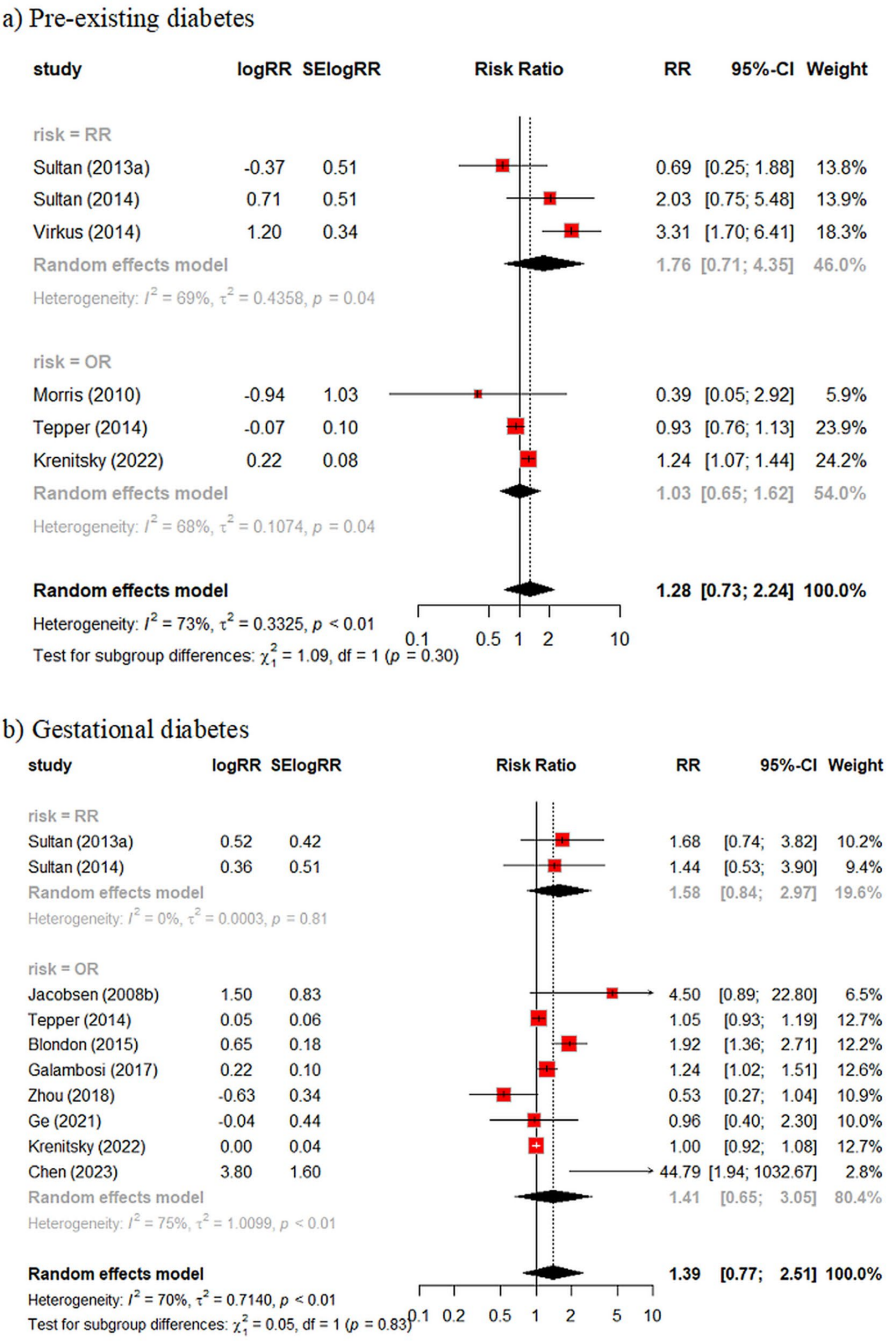
Diabetes and the risk of postpartum VTE. (a) Pre‐existing diabetes and (b) gestational diabetes.

Six studies provided data on pre‐existing diabetes and the risk of postpartum VTE (Figure 3). All were from cohort or cross‐sectional studies and used administrative databases. Overall, no clear relationship was observed with considerable between‐study heterogeneity (RR = 1.28, 95% CI 0.73 – 2.24, *I* ^2^= 73%). In a sensitivity analysis where the paper by Wen [36] instead of Krenitsky [28] was used to represent data from the National Readmissions database, the impact on the pooled estimate was marginal (RR = 1.21; 95% CI 0.68 – 2.15, *I* ^2^= 69%).

Ten studies provided data on gestational diabetes and the risk of postpartum VTE. While some studies reported an increase in risk in women with GD, the results were inconsistent with no overall evidence for an association (RR = 1.39, 95% CI 0.77 – 2.51, *I* ^2^= 70%).

### 3.6 Results: Antepartum and Postpartum Combined

**FIGURE 4:**
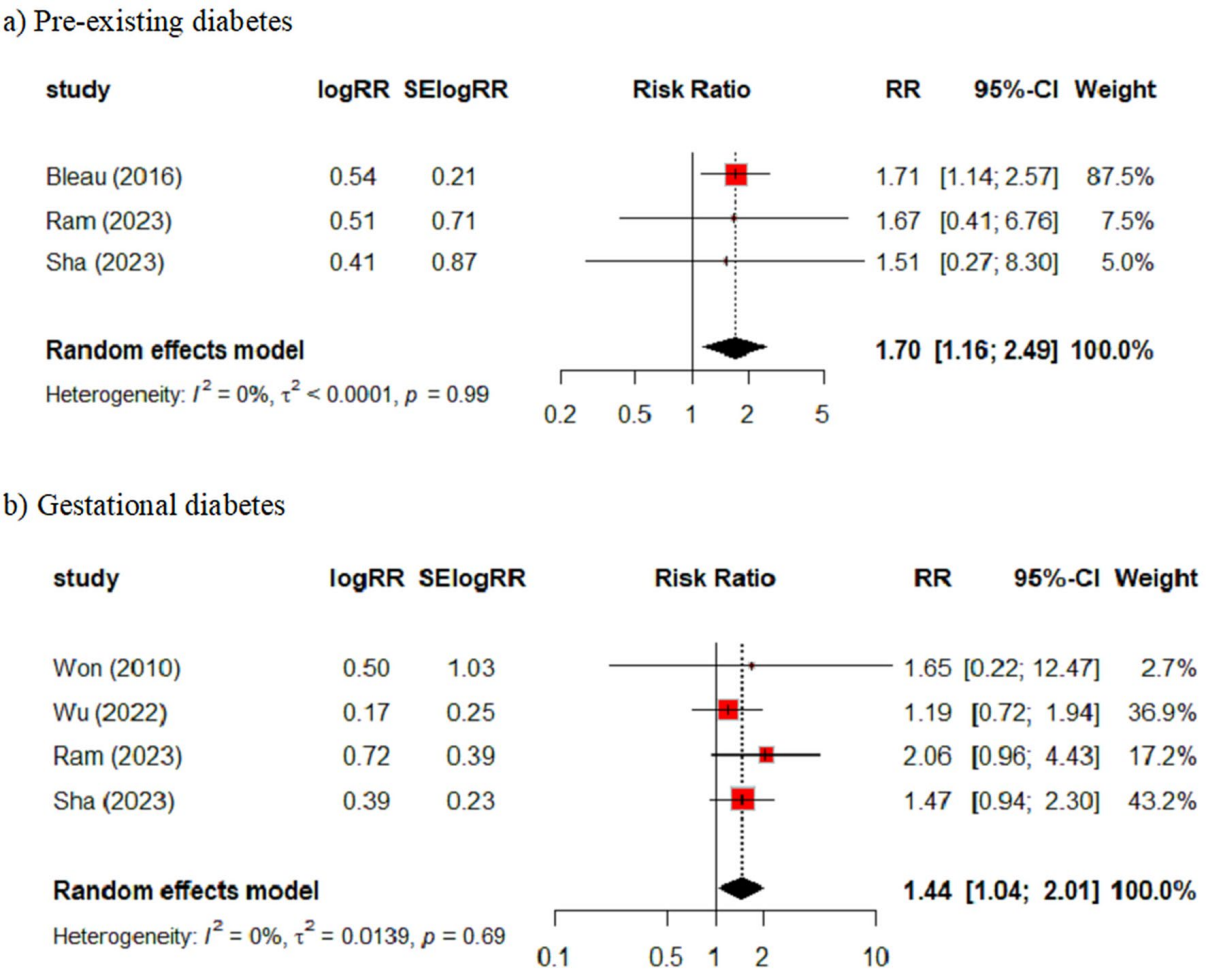
Diabetes and the risk of VTE in the antepartum and postpartum combined (entire maternity). (a) Pre‐existing diabetes and (b) gestational diabetes.

When the entire maternity was considered, three studies provided data on pre‐existing diabetes which when pooled showed a small increase in the risk of VTE with a consistent result between studies (RR = 1.70, 95% CI 1.16 – 2.49, *I* ^2^= 0%) (Figure 4). For gestational diabetes, four studies provided data, again highlighting a small increase in risk when results were pooled (RR = 1.44, 95% CI 1.04 – 2.01, *I* ^2^= 0%).

### 3.7 Sensitivity Analysis: Adjustment for BMI and Obesity in Analysis

Of the 21 studies included in one or more meta‐analysis, only six adjusted for a measure of BMI or obesity (Table S4). This made it difficult to ascertain the impact on results on making this restriction. One study adjusting for categorised BMI, reported an increased risk of antepartum VTE in women with pre‐existing diabetes [33]. There remained no evidence of increased risk associated with pre‐existing or gestational diabetes with postpartum VTE when restricted to studies adjusting for BMI or obesity. None of the five studies which provided data on VTE over the entire maternity were included in this sensitivity analysis.

## 4 Discussion

### 4.1 Main Findings

In a comprehensive review of literature using observational designs, we found no clear evidence of a link between diabetes and risk of VTE either during pregnancy or following childbirth. Individual studies reported higher risks of VTE during pregnancy specifically for both pre‐existing and gestational diabetes. However, heterogeneity between results was high. This would be expected based on differences in geography, study design, data source, methods of ascertaining VTE, differences in methodological quality and variables adjusted for in the analysis. We addressed the last of these by restricting analyses to studies which adjusted for a measure of BMI or obesity. From this there was no obvious suggestion that there was an association between pre‐existing or gestational diabetes and VTE risk which was mediated through BMI.

### 4.2 Interpretation (In Light of Other Evidence)

Our review of studies on postpartum VTE focussed on risk in the time immediately following childbirth with all but one study including only events within 3 months of delivery. This contrasts with a recent systematic review on the association between gestational diabetes and a number of cardiovascular and cerebrovascular outcomes which included VTE, which specifically focussed on long‐term follow‐up [40]. In a pooled analysis of four studies, they found a small but statistically significant 28% increase in VTE risk among women with gestational diabetes (RR = 1.28; 95% CI 1.13 – 1.46, *I* ^2^= 33%). In our equivalent analysis, pooling 10 studies on gestational diabetes and VTE postpartum we obtained a pooled RR of a similar magnitude but which had a wide confidence interval owing to both the small size of some of the studies being pooled and very high levels of heterogeneity (RR = 1.39; 95% CI 0.77 – 2.51; *I* ^2^= 70%). In the previous review, the increase in risk for VTE resulting from gestational diabetes was lower in magnitude than for other outcomes considered including stroke and any cardiovascular disease. They also found that the result was attenuated when restricting to studies with gestational diabetes but no subsequent diabetes. This analysis did not include any studies with VTE as an outcome, but could support suggestions that diabetes occurring specifically during pregnancy may not influence either short‐ or long‐term risk of serious cardiovascular outcomes if this resolves following childbirth. An earlier systematic review also found gestational diabetes to raise the risk of cardiovascular disease long term, but did not specifically include VTE as an outcome [41]. Diabetes was considered in a narrative review on this topic containing studies up to 2014 [42]. The conclusion from this work that thromboprophylaxis should only be considered where diabetes co‐exists with other risk factors is largely supported by our findings [42].

### 4.3 Strengths and Limitations

Our work has several strengths. This is the first study which has comprehensively reviewed all previous epidemiological evidence on the link between pre‐existing and gestational diabetes and maternal VTE, with no restrictions placed on geography or date of publication. Robust statistical methods were used to pool results from similar studies. Also, by stratifying results according to whether diabetes was pregestational or diabetes and providing separate analyses for antepartum and postpartum VTE (where available), our results have greater relevance for policy makers and clinicians.

This work also had limitations. First, the quality of any systematic review is only as good as the studies which contribute to this. Our studies were of observational design and two components in our quality assessment scored negatively for around one half of studies. Many studies did not identify and exclude women who had VTE previously. However, given the young age of the target population and rarity of the outcome this is unlikely to have biased findings. Also, there were differences in methods of ascertaining VTE between studies, with some registry studies relying solely on clinician codes. Second, this specific review was complicated through some databases such as the UK CPRD, the US Healthcare Cost and Utilisation Project Nationwide Readmissions Database and the Danish National Registry data all contributing more than one study. This required care in study selection and data extraction to avoid duplication of data. However, some duplication may be unavoidable especially, due to potential overlap in participants contributing to the CPRD and THIN databases as both used the same practice management software over the period these studies were conducted. The degree of overlap is unknown as information on participating practices is confidential, but notable differences in effect sizes from THIN and CPRD data included in the same meta‐analysis would indicate this overlap is unlikely to be substantial. The NOS we used to assess methodological quality may not completely capture the full RoB of a particular study, and some researchers have highlighted limitations with its use [43]. We chose this tool because we felt choice of confounder selection was the most important internal threat to validity and the NOS gave us flexibility to rate studies based on adjustment for covariates which we believed were crucial. Furthermore, given the importance of obesity in potentially explaining any association, we conducted additional sensitivity analysis restricted only to studies which took account of this factor. Finally, with only a modest number of studies contributing to any one meta‐analysis and with the absence of individual patient data, we were unable to comprehensively explore reasons for heterogeneity in findings.

## 5 Conclusion

In a comprehensive systematic review, we found no clear evidence of a link between either pre‐existing or gestational diabetes and the risk of maternal VTE, with high heterogeneity in results between studies. These results should enable healthcare workers to provide a degree of re‐assurance when counselling pregnant women with diabetes about their likely risk of VTE, giving attention to other risk factors which may co‐exist with this. However, the wide confidence intervals surrounding pooled effect estimates in this review will limit this degree of re‐assurance. The UK RCOG thromboprophylaxis guidelines include Type 1 diabetes with nephropathy as a risk factor which would make them eligible for both antepartum and postpartum VTE prophylaxis [6]. Another clinical guideline from Canada includes gestational diabetes in its guideline for postpartum thromboprophylaxis if it occurs alongside at least one other risk factor, [44] while the American College of Chest Physicians pregnancy and VTE guidelines make no specific mention of diabetes [45]. Data on Type 1 pre‐existing diabetes specifically was only provided for three of the studies in this review [20, 25, 26]. With registry‐based studies in particular rarely making this distinction because of how diabetes is coded, it may be difficult even for future studies to look at diabetes types separately to inform future guideline updates. Furthermore, studies included in this review did not consider the role of nephropathy, however, proteinuria has been found to be higher in women with a history of diabetes as well as being associated with VTE in pregnancy and postpartum [46]. This would therefore be a plausible explanation as to why VTE risk could be higher in women with a history of diabetes.

Future research on this topic should shed light on the heterogeneity of findings reported to date, in particular whether characteristics particular to a small number of studies which observed a large association between a reporting of diabetes and VTE may give more precise clues as to the nature of any such association [25, 33, 34, 35]. It is essential that future cohort studies on this topic are based on a causal inference framework which considers that postpartum VTE in particular is influenced by a large number of often highly correlated factors [47]. In this case, the value will be understanding the degree to which either pre‐existing or gestational diabetes may increase the risk of other risk factors, especially related to delivery which may themselves increase the risk of VTE [32]. Finally, observational studies can only go so far in helping us settle controversies in this area. It is important that future clinical trials and value of information studies are conducted focussing on thromboprophylaxis targeted towards women at highest risk [11]. Those at highest risk are where the benefits of receiving prophylaxis are considered to outweigh the harms according to various international guidelines [6, 44, 48]. In this review, we found insufficient evidence to advise that diabetes should be considered independently of obesity when identifying such women.

## Author Contributions

M.O. and M.J.G. were responsible for the conception and design of the study. M.O., E.B. and M.J.G. were responsible for screening of studies (titles, abstracts and full texts). M.O. and M.J.G. were responsible for data extraction and quality assessment. M.O. carried out the data analysis. M.O. and M.J.G. produced the first draft of the manuscript. All authors were responsible for critical evaluation of the manuscript. All authors accept responsibility for the paper as published.

## Ethics Statement

The authors have nothing to report.

## Conflicts of Interest

The authors declare no conflicts of interest.

## Supporting information


Data S1.

## Data Availability

The data that support the findings of this study are available from the corresponding author upon reasonable request.
