# Supplementary material for: Pre‐Existing and Gestational Diabetes and Risk of Maternal Venous Thromboembolism: A Systematic Review and Meta‐Analysis of Observational Studies
Source: BJOG. 2024 Dec 17;132(8):1076–85. doi: 10.1111/1471-0528.18043 (PMC12137783; doi:10.1111/1471-0528.18043)
Supplement: Supplementary file 1 — Data S1. [file BJO-132-1076-s001.docx]

Pre-existing and gestational diabetes and risk of maternal venous thromboembolism: A systematic review and meta-analysis of observational studies

Supporting Information

**Appendix S1: Search Strategy**

Medline (Ovid)

1. exp Venous Thrombosis/
2. exp Venous Thromboembolism/
3. exp Thrombosis/
4. Pulmonary Embolism.mp. and exp Pulmonary Embolism/
5. (dvt* or (deep$ adj8 (vein$ or ven$) adj8 thromb$) or embol$).mp. [mp=title, original title, abstract, name of substance word, subject heading word, unique identifier]
6. 1 or 2 or 3 or 4 or 5
7. exp Pregnancy/
8. Pregnancies.mp.
9. Antepartum.mp.
10. exp Postpartum Period/
11. Postpartum.mp.
12. Gestation*.mp.
13. exp Pregnancy Outcome/
14. Pregnancy outcomes.mp.
15. Outcome, Pregnancy.mp.
16. exp Pregnancy, High-Risk/
17. High-Risk Pregnancies.mp.
18. 7 or 8 or 9 or 10 or 11 or 12 or 13 or 14 or 15 or 16 or 17
19. exp Gestational Diabetes
20. exp Diabetes Complications/
21. exp Diabetes, Gestational/
22. exp Diabetes Mellitus/
23. exp Diabetes Mellitus, Type 1/
24. exp Diabetes Mellitus, Type 2/
25. Diabet*.mp.
26. Exp Pregnancy in Diabetics/
27. Pre-existing diabetes.mp.
28. Risk factor*.mp.
29. Risk Factor/
30. 20 or 21 or 22 or 23 or 24 or 25 or 26 or 27 or 28 or 29
31. 18 or 19
32. 6 and 30 and 31

Embase (Ovid)

1. exp Venous Thrombosis/
2. exp Venous Thromboembolism/
3. exp Thrombosis/
4. Pulmonary Embolism.mp. and exp Pulmonary Embolism/
5. (dvt* or (deep$ adj8 (vein$ or ven$) adj8 thromb$) or embol$).mp. [mp=title, original title, abstract, name of substance word, subject heading word, unique identifier]
6. 1 or 2 or 3 or 4 or 5
7. exp Pregnancy/
8. Pregnancies.mp.
9. Antepartum.mp.
10. exp Postpartum Period/
11. Postpartum.mp.
12. Gestation*.mp.
13. exp Pregnancy Outcome/
14. Pregnancy outcomes.mp.
15. Outcome, Pregnancy.mp.
16. exp Pregnancy, High-Risk/
17. High-Risk Pregnancies.mp.
18. 7 or 8 or 9 or 10 or 11 or 12 or 13 or 14 or 15 or 16 or 17
19. exp Diabetes Complications/
20. exp Diabetes, Gestational/
21. exp Diabetes Mellitus/
22. exp Diabetes Mellitus, Type 1/
23. exp Diabetes Mellitus, Type 2/
24. Diabet*.mp.
25. Exp Pregnancy in Diabetics/
26. Pre-existing diabetes.mp.
27. Risk factor*.mp.
28. Risk Factor/
29. 19 or 20 or 21 or 22 or 23 or 24 or 25 or 26 or 27 or 28
30. 6 and 18 and 29
31. conference abstract.pt.
32. 30 not 31

Google Scholar

Search phrase “Risk of Venous Thromboembolism in Pregnant and Postpartum Women with Pre-Existing or Gestational Diabetes”

The first 300 results when sorted by relevance were included in the primary search.

**Appendix S2: Newcastle - Ottawa Quality Assessment Scale**

(A) Case-Control Studies

Note: A study can be awarded a maximum of one star for each numbered item within the Selection and Exposure categories. A maximum of two stars can be given for Comparability.

**Selection**

1) Is the case definition adequate?

a) yes, with independent validation *****

b) yes, eg record linkage or based on self reports

c) no description

2) Representativeness of the cases

a) consecutive or obviously representative series of cases *****

b) potential for selection biases or not stated

3) Selection of Controls

a) community controls *****

b) hospital controls

c) no description

4) Definition of Controls

a) no history of disease (endpoint) *****

b) no description of source

**Comparability**

1) Comparability of cases and controls on the basis of the design or analysis

a) study controls for _______________ (Select the most important factor.) *****

b) study controls for any additional factor ***** (This criteria could be modified to indicate specific control for a second important factor.)

**Exposure**

1) Ascertainment of exposure

a) secure record (eg surgical records) *****

b) structured interview where blind to case/control status *****

c) interview not blinded to case/control status

d) written self report or medical record only

e) no description

2) Same method of ascertainment for cases and controls

a) yes *****

b) no

3) Non-Response rate

a) same rate for both groups *****

b) non respondents described

c) rate different and no designation

(B) Cohort and Cross-Sectional Studies

**Selection**

1) Representativeness of the exposed cohort

a) truly representative of the average __________ (describe) in the community *****

b) somewhat representative of the average _________ in the community *****

c) selected group of users eg nurses, volunteers

d) no description of the derivation of the cohort

2) Selection of the non exposed cohort

a) drawn from the same community as the exposed cohort *****

b) drawn from a different source

c) no description of the derivation of the non exposed cohort

3) Ascertainment of exposure

a) secure record (eg surgical records) *****

b) structured interview *****

c) written self report

d) no description

4) Demonstration that outcome of interest was not present at start of study

a) yes *****

b) no

**Comparability**

1) Comparability of cohorts on the basis of the design or analysis

a) study controls for _____________ (select the most important factor) *****

b) study controls for any additional factor ***** (This criteria could be modified to indicate specific control for a second important factor.)

**Outcome**

1) Assessment of outcome

a) independent blind assessment *****

b) record linkage *****

c) self report

d) no description

2) Was follow-up long enough for outcomes to occur

a) yes (select an adequate follow up period for outcome of interest) *****

b) no

3) Adequacy of follow up of cohorts

a) complete follow up - all subjects accounted for *****

b) subjects lost to follow up unlikely to introduce bias - small number lost - > ____ % (select an adequate %) follow up, or description provided of those lost) *****

c) follow up rate < ___% (select an adequate %) and no description of those lost

d) no statement

**Table S1.**

*Characteristics of Included Studies*

| Author and Year | Country | Study design | Sample size^†^ | Data source | Study Population | Postpartum duration | Exposure | Ascertainment of VTE (DVT and/or PE) |
| --- | --- | --- | --- | --- | --- | --- | --- | --- |
| Bleau 2016^37^ | United States | Cross sectional | 7,917,453 | Health Care Cost and Utilization Project, Nationwide Inpatient Sample (HCUP-NIS)  Study conducted between 2003 and 2011 | Antepartum and postpartum  (combined data only) | 6 weeks | Pre-existing diabetes. ICD-9-CM code – 250 fifth digit 1 | Diagnosis of deep vein thrombosis (DVT), pulmonary embolism (PE), or both using ICD-9 codes |
| Blondon 2015^36^ | United States | Case-Control | 547 cases, 9482 controls | Registry of all births in Washington State  Study conducted between Jan 1987 and Dec 2011 | Postpartum | 3 months | Gestational diabetes  codes not provided | ICD-9-CM  code for DVT or PE at a delivery hospitalization or during the postpartum  period.  (validation study yielded a PPV of 90% for PE codes and 78% for DVT codes) |
| Chen 2023^38^ | China | Case-Control | 384 cases, 768 controls  Cases and controls did not experience anticoagulant treatment during the perinatal period | Women who delivered in 12 hospitals throughout China.  Study conducted between Jan 2019 and Jan 2022 | Postpartum | Delivery hospitalisation | Gestational diabetes  codes not provided | DVT of the lower extremity was con­firmed by color Doppler ultrasonography. PE was confirmed by pulmonary CT examination. |
| Galambosi 2017^35^ | Finland | Cohort | 634,292 women | Care Register for Health Care (HILMO), the National Medical Birth Register and the Register of Induced Abortions, Cause of Death Register and Population Register Center  Study conducted between 2001 and 2011 | Postpartum women | 180 days | Gestational diabetes  codes not provided | Women with an ICD-  10 code of DVT (I80.1–I80.9), portal vein thrombosis  (I82.0–I82.9), PE (I26.0, I26.9) and VTE specifically related to postpartum or delivery  (O87.1 and O88.2) |
| Ge 2021^34^ | China | Case-Control | 38 cases, 152 controls  Stated that very few women received pharmacological prophylaxis. | International Peace Maternity and Child Health Hospital, Shanghai  Study conducted between June 2016 and June 2020. | Postpartum | Not specified | Gestational diabetes  codes not provided | The diagnosis of DVT was confirmed by compression ultraso­nography of the lower limb veins. Presence of PE was checked using  echocardiography and computer tomography pulmonary an­giography |
| Jacobsen 2008a^32^ | Norway | Case-Control | 301 cases, 613,232 controls | Norwegian Medical Birth Registry  Study conducted between 1990 and 2003 | Antepartum | NA | Gestational diabetes  codes not provided | ICD 8, 9 and 10 codes related to VTE. Each case validated using hospital records. Definite  Objective diagnosis of DVT was confirmed by Compression or color Doppler ultrasonography or by venography. PE was confirmed by perfusion lung scanning, computed tomography, magnetic resonance imagery or angiography. |
| Jacobsen 2008b^33^ | Norway | Case-Control | 291 cases, 1229 controls | Norwegian Medical Birth Registry  Study conducted between 1990 and 2003 | Postpartum | 3 months | Gestational diabetes  codes not provided | ICD 8, 9 and 10 codes related to VTE. Each case validated using hospital records. Definite  Objective diagnosis of DVT was confirmed by Compression or color Doppler ultrasonography or by venography. PE was confirmed by perfusion lung scanning, computed tomography, magnetic resonance imagery or angiography. |
| Jensen 2013^31^ | Denmark | Cohort | 299, 180 pregnancies | The Medical Birth Registry, Civil Registration System and National Patient Register  Study conducted between 2003 and 2010 | Antepartum | NA | Gestational diabetes  ICD-10 code O244 | ICD-10 codes for deep vein thrombosis and pulmonary embolism. |
| Krenitsky 2022^30^ | United States | Cross-sectional | 73,109,789 deliveries | The National Inpatient Sample from the Healthcare Cost and Utilization Project  Study conducted between 2000 and 2018. | Postpartum | Delivery hospitalisation | Pre-existing and gestational diabetes  codes not provided | Identified  using ICD-10-CM diagnosis codes from a review of the  literature and prior studies |
| Morris 2010^29^ | Australia | Cohort | 510,889 pregnancies | Database of hospitals in New South Wales  Study conducted between 2001 and 2006 | Postpartum | 12 weeks | Pre-existing  codes not provided | Pulmonary embolism in first 10 ICD-10 diagnosis fields, codes O88.2, I26.9, I82.2. |
| Ram 2023^28^ | Israel | Cohort | 421,125 live births  Women who purchased anticoagulant drugs during pregnancy or postpartum were excluded. | Digital  database of Maccabi Healthcare Services, an integrated Health  Maintenance Organization (HMO)  Study conducted between 2010 and 2019 | Antepartum and Postpartum (combined data only) | 6 weeks | Pre-existing and gestational diabetes  codes not provided | ICD-9-CM codes for DVT (453.4, 453.41, 453.42, 453.8) and PE (415.1). |
| Sha 2023^27^ | China | Case-Control | 197 cases, 591 controls | Department of Obstetrics, Tongji Hospital of Tongji Medical College of Huazhong in Wuhan  Study conducted between Jan 2010 and June 2022 | Antepartum and Postpartum (combined data only) | 6 weeks | Pre-existing and gestational diabetes  codes not provided | DVT in patients with clinical manifestations was objectively confirmed by compression  or color Doppler ultrasonography and PE was diagnosed  through ventilation–perfusion scan or computed tomography pulmonary angiography (CTPA). |
| Sultan 2013a^25^ | United Kingdom | Cohort | 280,451  0.4% received prophylaxis based on primary care data only (no secondary care data available) | The Health Improvement Network (THIN)  Study conducted between Jan 1995 and July 2009 | Antepartum and postpartum | 12 weeks | Pre-existing and gestational diabetes  ICD-10 codes pre-existing E100-E149, O240-O243 Gestational O244,O249 | Read code from physician providing this was supported by either:  i)Evidence of anticoagulant treatment/ therapy within 90 days of diagnosis  ii)Death within 30 days of diagnosis |
| Sultan 2013b^24^ | England | Cohort | 206,785 | Clinical Practice Research Datalink (CPRD) linked to Hospital Episodes Statistics (HES)  Study conducted between 1997 and 2010 | Antepartum | NA | Gestational diabetes  ICD-10 codes O244, O249 | Medical code from physician for PE or DVT in HES or CPRD, provided this was supported by:  i)Evidence of anticoagulant treatment/ therapy within 90 days of diagnosis  ii)Death within 30 days of diagnosis |
| Sultan 2014^26^ | England | Cohort | 160,007 | Clinical Practice Research Datalink (CPRD) linked to Hospital Episodes Statistics (HES)  Study conducted between 1997 and 2010 | Postpartum | 6 weeks | Pre-existing and gestational diabetes ICD-10 codes pre-existing E100-E149, O240-O243 Gestational O244,O249 | A Read or ICD-10 diagnosis codes providing this was supported by either:  i) Evidence of anticoagulant treatment/ therapy within 90 days of diagnosis  iii)Death within 30 days of diagnosis |
| Tepper 2014^14^ | United States | Cross- sectional | 2,541,562  (Separate data for private medical care, n=1,540,026 and Medicaid, n=1,001,536)  Women who were prescribed anticoagulants in the 6 months preceding the delivery admission were excluded | Truven Health MarketScan Commercial and Multi-State Medicaid databases  Study conducted between 2005 and 2011. | Postpartum | 12 weeks | Pre-existing and gestational diabetes  ICD-9-CM codes – Pre-existing 250.00–250.9, 648.0x  Gestational 648.8x | Evidence of anticoagulant treatment/ therapy within 90 days of diagnosis using  ICD-9-CM Codes |
| Virkus 2014^23^ | Denmark | Cohort | 1,297,037  0.1% of VTE cases received anticoagulant prophylaxis treatment | The Central Person Registry, The National Registry of Patients, The Danish Cause of Death Registry, Statistics of Denmark, The National Registry of Medical Products Statistics, The Danish Birth Registry  Study conducted between January 1995 and December 2009 | Antepartum and postpartum | 12 weeks | Pre-existing diabetes  ICD-10 codes – E100-E149 | Ultrasonography, venography, ventilation-perfusion lung scan, computer-tomography, or a magnetic resonance scan.  Evidence of anticoagulant treatment/ therapy for the rest of pregnancy or puerperal period, or for at least three months |
| Wen 2018^22^ | United states | Cross- sectional | 6,269,641 | Healthcare Cost and Utilization Project Nationwide Readmissions Database (NRD)  Study conducted in 2013 and 2014. | Postpartum | 60 days | Pre-existing diabetes  codes not provided | ICD-9-CM codes for either acute DVT or PE. |
| Won 2010^21^ | South Korea | Cross- sectional | 57,009 | Cheil General Hospital  Study conducted between Jan 2002 and Dec 2008. | Antepartum and Postpartum (combined data only) | 4 weeks | Gestational diabetes  Diagnosed when glucose levels exceed ≥2 of fasting 95 mg/dL; 1 hour 180 mg/dL; 2 hours 155 mg/dL; 3 hours, 140 mg/dL. | DVT was objectively confirmed by an intraluminal filling defect and non-compression viewed by colour Doppler ultrasonography.  PE was confirmed by observation of an intraluminal filling defect on computed tomography pulmonary angiography. |
| Wu 2022^20^ | China | Case-control | 161 cases, 1610 controls | Shanghai First Maternity  and Infant Hospital  Study conducted between Jan 2017 and Sep 2021 | Antepartum and Postpartum (combined data only) | 6 weeks | Gestational diabetes codes not provided | Search for ICD-9 or ICD-10 codes using the hospital information system. |
| Zhou 2018^19^ | China | Case-control: | 102 cases, 408 controls | Women’s Hospital, Zhejiang  Study conducted between Jan 2006 and Dec 2016 | Postpartum | 1 week | Gestational diabetes  codes not provided | Recorded medical code from a physician within 1 week of childbirth. Supplemented by evidence of Vascular ultrasound or computed tomography angiography. |

^†^ Where data on any VTE prophylaxis with anticaogulant medications during the study period is reported in the paper, this is included under “Sample size”. Where blank, this means either no mention is made of VTE prophyalxis or it is specifically mentioned that these data are not available.

**Table S2**

*Critical Appraisal of Included Studies, Using the Newcastle-Ottawa Scale*

|  | No. of Stars | | | |
| --- | --- | --- | --- | --- |
| Author and Year | Selection | Comparability | Outcome^†^/Exposure^‡^ | Overall Score* |
| Bleau 2016^37^ | 3 | 1 | 2 | 6 |
| Blondon 2015^36^ | 4 | 0 | 2 | 6 |
| Chen 2023^38^ | 4 | 2 | 2 | 8 |
| Galambosi 2017^35^ | 3 | 1 | 2 | 6 |
| Ge 2021^34^ | 3 | 0 | 3 | 6 |
| Jacobsen 2008a^32^ | 4 | 2 | 3 | 9 |
| Jacobsen 2008b^33^ | 4 | 2 | 3 | 9 |
| Jensen 2013^31^ | 3 | 2 | 2 | 7 |
| Krenitsky 2022^30^ | 3 | 2 | 1 | 6 |
| Morris 2010^29^ | 3 | 2 | 2 | 7 |
| Ram 2023^28^ | 3 | 0 | 2 | 5 |
| Sha 2023^27^ | 3 | 0 | 3 | 6 |
| Sultan 2013a^25^ | 4 | 2 | 3 | 9 |
| Sultan 2013b^24^ | 4 | 0 | 3 | 7 |
| Sultan 2014^26^ | 4 | 0 | 3 | 7 |
| Tepper 2014^14^ | 3 | 2 | 3 | 8 |
| Virkus 2014^23^ | 4 | 0 | 3 | 7 |
| Wen 2018^22^ | 4 | 2 | 2 | 8 |
| Won 2010^21^ | 3 | 2 | 2 | 7 |
| Wu 2022^20^ | 3 | 0 | 3 | 6 |
| Zhou 2018^19^ | 3 | 0 | 3 | 6 |

^†^For cohort and cross sectional studies

^‡^For case-control studies

*Maximum score 9

**Table S3.**

*Effect Measures, Statistical Techniques, and Covariates in Included Studies.*

| Author and Year | Effect Measure | Model | Covariates |
| --- | --- | --- | --- |
| Bleau 2016^37^ | OR | Logistic regression | Maternal age |
| Blondon 2015^36^ | OR | Logistic regression | None |
| Chen 2023^38^ | OR | Logistic regression | Age, BMI, mode of delivery, family history of diabetes or cardiovascular disease, no. of deliveries, assisted fertilisation, and D-dimer before delivery |
| Galambosi 2017^35^ | OR | Logistic regression | Maternal age, mode of delivery, year of delivery, and thrombophilia. |
| Ge 2021^34^ | OR | Chi-square | None |
| Jacobsen 2008a^32^ | OR | Logistic regression | Age, parity, premature rupture, multiple pregnancy, and assisted reproduction |
| Jacobsen 2008b^33^ | OR | Logistic regression | Age, parity, smoking, weight gain, pre-eclampsia, premature rupture of membranes, BMI, method of conception. multiple pregnancy, mode of delivery, postpartum infection, pre-postpartum bleeding, and postpartum surgery |
| Jensen 2013^31^ | HR | Cox regression | BMI, smoking, previous VTE, previous stroke, vascular disease, thrombophilia, gestational hypertension, hyperemesis, gestational diabetes, pre-eclampsia, and calendar time |
| Krenitsky 2022^30^ | OR | Logistic regression | Maternal race, age, payer type, income quartile, hypertensive disorders of pregnancy, chronic hypertension, mode of delivery, postpartum haemorrhage, transfusion, postpartum infection, obesity, asthma. stillbirth, preterm delivery, smoking, chronic heart disease, multiple gestation, history of VTE, thrombophilia and year. |
| Morris 2010^29^ | OR | Logistic regression | Age, parity, multiple gestation, gestational hypertension, lupus, prior fetal loss, prior stillbirth, caesarean delivery, preterm birth, birth weight, transfusion, public/private hospital status |
| Ram 2023^28^ | OR | Logistic regression | None |
| Sha 2023^27^ | OR | Logistic regression | None |
| Sultan 2013a^25^ | IRR | Poisson regression | Antepartum: adjusted for: maternal age, parity, BMI, smoking status.  Postpartum: adjusted for: maternal age, parity, BMI, mode of delivery, pregnancy length, obstetric haemorrhage, varicose veins, IBD, cardiac disease, smoking status. |
| Sultan 2013b^24^ | IRR | Poisson regression | None |
| Sultan 2014^26^ | IRR | Poisson regression | None |
| Tepper 2014^14^ | OR | Logistic regression | Maternal age, race, obesity, smoking, chronic hypertension, preeclampsia, mode of delivery, multiple birth, anaemia, antepartum haemorrhage, postpartum haemorrhage, postpartum infection, heart failure, pulmonary oedema, adult respiratory distress syndrome, disseminated intravascular coagulation, shock, sepsis, cerebrovascular disorders, ventilation, peripartum cardiomyopathy, cardiovascular conditions (including myocardial infarction). |
| Virkus 2014^23^ | IRR | Poisson regression | None |
| Wen 2018^22^ | OR | Logistic regression | Maternal age, income, insurance, thrombophilia, tobacco, history of VTE, hypertension, caesarean delivery, prolonged stay, postpartum haemorrhage with transfusion, infection, multiple gestation, hospital teaching, hospital location, hospital bed size. |
| Won 2010^21^ | OR | Logistic regression | Maternal age, caesarean section, multiple pregnancies, pregnancy-induced hypertension, assisted reproduction technology, placenta previa, placenta abruption. |
| Wu 2022^20^ | OR | Logistic regression | None |
| Zhou 2018^19^ | OR | Logistic regression | None |

HR Hazard ratio; OR Odds ratio; IRR Incidence rate ratio

**Table S4**

*Sensitivity analysis pooling only studies which adjusted for BMI or a measure of obesity*

|  | All studies | | | Studies adjusting for BMI or obesity | | |
| --- | --- | --- | --- | --- | --- | --- |
|  | No. of studies | Pooled Risk Ratio (95% CI) | I^2^ (%) | No. of studies | Pooled Risk Ratio (95% CI) | I^2^ (%) |
| 1) Antepartum: pre-existing | 2 | 1.71 (0.43, 6.77) | 68 | 1 | 3.54 (1.13, 11.0) | - |
| 2) Antepartum: gestational | 4 | 2.48 (1.47, 4.16) | 45 | 2 | 1.56 (0.92, 2.64) | 0 |
| 3) Postpartum: pre-existing | 6 | 1.28 (0.73, 2.24) | 73 | 3 | 1.05 (0.80, 1.38) | 67 |
| 4) Postpartum: gestational | 10 | 1.39 (0.77, 2.51) | 70 | 5 | 2.08 (0.64, 6.73) | 62 |
| 5) Combined: pre-existing | 3 | 1.70 (1.16, 2.49) | 0 | 0 | - | - |
| 6) Combined: gestational | 4 | 1.44 (1.04, 2.01) | 0 | 0 | - | - |
